# Efficient and effective single-step screening of individual samples for SARS-CoV-2 RNA using multi-dimensional pooling and Bayesian inference

**DOI:** 10.1098/rsif.2021.0155

**Published:** 2021-06-16

**Authors:** Juliana Sobczyk, Michael T. Pyne, Adam Barker, Jeanmarie Mayer, Kimberly E. Hanson, Matthew H. Samore, Rodrigo Noriega

**Affiliations:** ^1^ Department of Pathology, University of Utah School of Medicine, Salt Lake City, UT, USA; ^2^ ARUP Institute for Clinical and Experimental Pathology®, Salt Lake City, UT, USA; ^3^ Division of Epidemiology, University of Utah Health Sciences Center, Salt Lake City, UT, USA; ^4^ Division of Infectious Diseases, University of Utah Health Sciences Center, Salt Lake City, UT, USA; ^5^ Division of Infectious Disease, University of Utah School of Medicine, Salt Lake City, UT, USA; ^6^ Division of Epidemiology, Department of Internal Medicine, University of Utah School of Medicine, Salt Lake City, UT, USA; ^7^ Informatics, Decision Enhancement, and Analytic Science (IDEAS) Center of Innovation, Veterans Affairs Salt Lake City Health Care System, Salt Lake City, UT, USA; ^8^ Department of Chemistry, University of Utah, Salt Lake City, UT, USA

**Keywords:** SARS-CoV-2, pooling, non-adaptive screening, Bayesian, RT-PCR, Monte Carlo

## Abstract

Rapid and widespread implementation of infectious disease surveillance is a critical component in the response to novel health threats. Molecular assays are the preferred method to detect a broad range of viral pathogens with high sensitivity and specificity. The implementation of molecular assay testing in a rapidly evolving public health emergency, such as the ongoing COVID-19 pandemic, can be hindered by resource availability or technical constraints. We present a screening strategy that is easily scaled up to support a sustained large volume of testing over long periods of time. This non-adaptive pooled-sample screening protocol employs Bayesian inference to yield a reportable outcome for each individual sample in a single testing step (no confirmation of positive results required). The proposed method is validated using clinical specimens tested using a real-time reverse transcription polymerase chain reaction test for SARS-CoV-2. This screening protocol has substantial advantages for its implementation, including higher sample throughput, faster time to results, no need to retrieve previously screened samples from storage to undergo retesting, and excellent performance of the algorithm's sensitivity and specificity compared with the individual test's metrics.

## 1. Introduction

Epidemiological strategies to control the spread of infectious respiratory diseases include contact, droplet and/or aerosol precautions, asymptomatic screening, contact tracing, case isolation, ring containment and social distancing [1–7]. While the specific set of strategies employed depends on the aetiological agent, the use of microbiological and molecular testing to identify disease cases is a crucially important element. In general, two key parameters for the performance of a diagnostic test are its *sensitivity* (a measure of its ability to correctly detect a positive case) and its *specificity* (a measure of its ability to only yield a positive result for the agent in question). Reducing the sensitivity of a test leads to an increase in false-negative results; a reduction in the specificity leads to an increase in false-positive-results. For practical purposes, other considerations for deploying diagnostics tests can include their ease of use, portability, cost and throughput.

The present COVID-19 pandemic has caused significant public health challenges, which have been compounded by resource constraints and the unprecedented scale of testing required to effectively control its spread [8–13]. To solve these challenges, sample pooling has been proposed as a means to scale up screening at a population level and to assess the prevalence of the disease [14–20]. However, currently available pooling protocols have significant limitations. First, the requirement of retesting individual samples for the identification of positive cases incurs substantial logistical challenges when screening a large number of samples at a constant rate for a prolonged period of time. Second, multi-dimensional pooling strategies that identify cases via the intersection of positive pools suffer from reduced performance when imperfect tests are used (i.e. sensitivity and/or specificity less than 100%). Finally, while high viral load samples can be diluted by pooling and still yield accurate test results, dilution of low viral load samples into large pools at low prevalence rates lowers the sensitivity of pooled tests [14]. Limiting the size of pools can reduce the effect of sensitivity loss, but it also reduces efficiency gains. Here, we report a screening strategy that employs multi-dimensional sample pooling and applies Bayesian inference to provide reportable results at the individual level in a single testing step. This strategy increases testing throughput with minimal or no loss of sensitivity over a reasonable range of disease prevalence. Importantly, this strategy is easily scalable to support a sustained large volume of testing over long periods of time.

**Figure 1.**
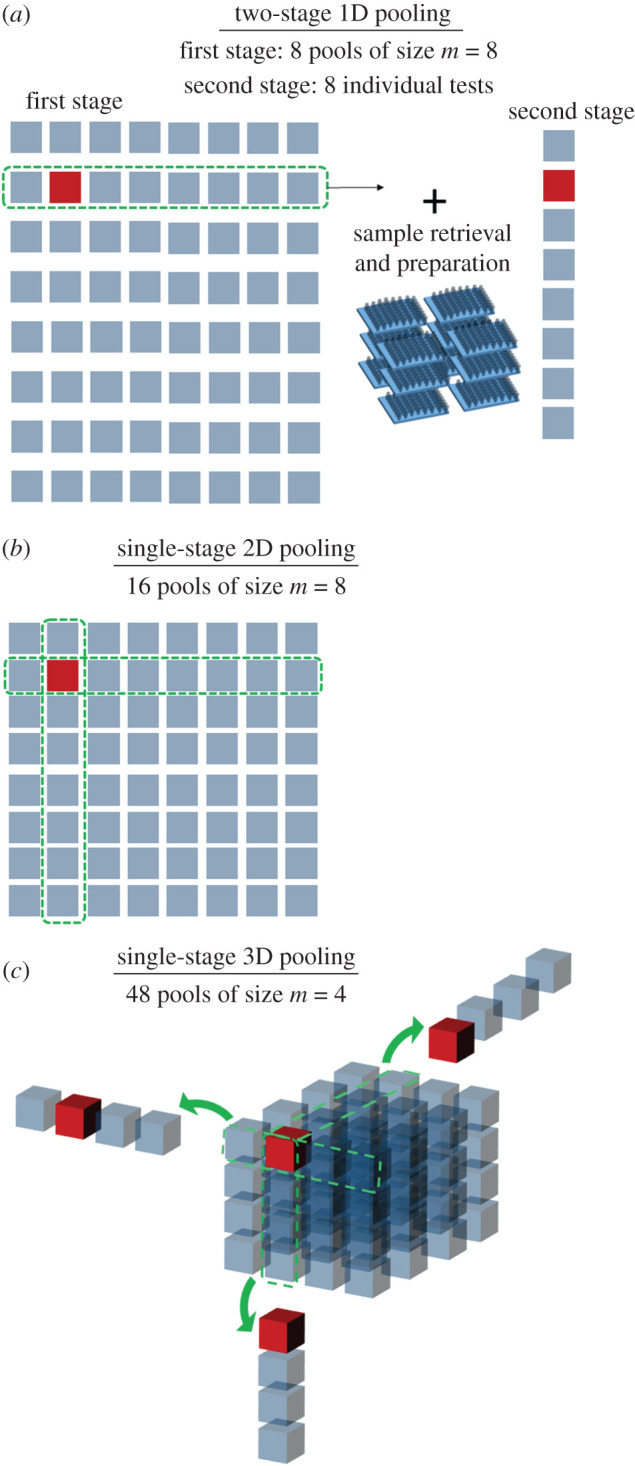
Visual representation of three pooled-sample approaches to screen 64 specimens, one of which is positive (marked in red), using (*a*) two-stage adaptive 1D pooling, (*b*) non-adaptive 2D pooling based on the intersection of positive pools and (*c*) non-adaptive 3D pooling with Bayesian inference. Pools that contain the positive specimen are highlighted, and in the case of 3D pooling, copied outside of the cube for clarity.

In standard two-stage one-dimensional (1D) sample pooling (figure 1*a*), specimens from $N_{s}$ patients are randomly pooled into groups of *m* samples each. Pooled samples are interrogated with the diagnostic test. If pooled testing yields a negative result, no further testing is conducted and all samples in that pool are assigned a negative result. If pooled testing yields a positive result, all patients in that pool are tested individually. This adaptive protocol, known as Dorfman pooling, was introduced in the 1940s, and subsequent developments include multiple levels of hierarchical testing where positive pools are divided into smaller pools until testing at the individual level is reached [21–26]. Adaptive pooling methods can yield considerable improvements in sample throughput, especially at low prevalence. However, large efficiency gains require large pool sizes and are affected by a reduction in sensitivity because of dilution. Some adaptive pooling strategies include an intermediate pool design step to maximize the information gained with each round of tests, increasing the algorithm efficiency [27]. Even without intermediate pool design steps, the logistical complications of retrieving samples belonging to positive pools and the need to undergo multiple rounds of testing can negatively impact the time required to identify positive specimens.

To circumvent the drawbacks of adaptive pooling, various non-adaptive pooling methods in multiple dimensions have been introduced (e.g. shifted transversal design) [28–31]. In a simple example of two-dimensional (2D) non-adaptive pooling, specimens are arranged in a matrix and samples are pooled along each row and along each column (figure 1*b*). Positive specimens are identified by the intersections of rows and columns that yield a positive test result, which requires close-to-ideal test characteristics. Non-adaptive screening methods have been developed to account for non-idealities [32], and similar tools used for DNA library screening have included Markov chain analysis and Bayesian probability estimation to interpret test results [33]. Here, we present a non-adaptive multi-dimensional pooling method that incorporates Bayesian inference to assess the probability of all individual specimens, and we explore the effects of dimensionality, pool size and test characteristics on its performance, efficiency and complexity.

## 2. Methods

The result of a diagnostic test can be evaluated with a Bayesian probability formalism [34–36]. In the context of an individual sample, this analysis takes into account the probability of detecting a positive case (sensitivity, Se) and the probability of a positive result from healthy samples (whose complement is the specificity, Sp). Bayesian inference requires the assessment of a prior probability for the presence of disease in a sample, P(D), which is updated to a posterior probability given a positive/negative test result with conditional probabilities P(D|+) or P(D|−), as shown in the following equations:

2.1a $$P(D|+)=\frac{P(D)\cdot \mathrm{Se}}{P_{\mathrm{pool}}(+)},$$

2.1b $$P(D|-)=\frac{P(D)\cdot (1-\mathrm{Se})}{1-P_{\mathrm{pool}}(+)},$$

where Se is the test's sensitivity and $P_{\mathrm{pool}}(+)$ is the overall probability of the pool yielding a positive test outcome. The probability of a positive result in a pooled sample is based on all prior probabilities for specimens in the pool. Upon extension to pooled-sample testing, a pool size-dependent reduction in test sensitivity is included by assuming that a fraction γ of true-positive specimens will be diluted below detectable levels due to pooling (details in electronic supplementary material, figure S1 and accompanying supplementary text). In this way, the probability of a positive test result in a pool is the sum of two contributions: the probability of having a non-zero number of detectable positive individual specimens multiplied by the test's sensitivity; and the probability of a sample without a single detectable positive specimen multiplied by the false-positive rate $P_{\mathrm{fp}}=1-\mathrm{Sp}$ (equation (2.2)):

2.2 $$P_{\mathrm{pool}}(+)=\left\{1-\prod_{i}[1-\gamma \cdot P_{i}(D)]\right\}\times \mathrm{Se}+(1-\mathrm{Sp})\times \prod_{i}[1-\gamma \cdot P_{i}(D)],$$

where *i* is a dummy index that runs over all samples belonging to the pool.

Here, we consider $N_{s}$ specimens arranged in a d-dimensional hypercube of size {*m*_1_, *m*_2_, …, *m_d_*}. Pools are constructed for all samples that form a line along a particular axis. Using the test outcome for each pooled sample, the posterior probabilities of all specimens belonging to it are calculated with a Bayesian inference step (equations (2.1*a*,*b*) and (2.2)). The posterior probability resulting from the application of equations (2.1*a*,*b*) and (2.2) to the test outcomes in each hypercube surface (i.e. from each pooling dimension) is used as a prior for the next round of posterior probability calculations along the next pooling dimension. Because of imperfect test characteristics and the possibility of multiple positive specimens belonging to the same pool at increased disease prevalence rates, not every surface of the hypercube contains the same number of positive test results. Therefore, the order in which we use the information contained in each of these surfaces can affect the posterior probability assigned to individual samples and thus their screening outcome. To account for this situation, we consider all d! permutations of the hypercube surfaces and include only those in which the number of positive tests follows an increasing (or flat) trend. For each one of such permutations, the posterior probability of all samples is calculated. The reported disease probability for each specimen is the largest value obtained among all the considered permutations (example distributions of posterior probabilities in electronic supplementary material, figures S2 and S3). A posterior probability threshold is used to assign a positive or negative screening result—lowering its value increases the algorithm sensitivity and reduces its specificity (electronic supplementary material, figures S4 and S5).

A three-dimensional (3D) example with equal pool size m=4 for all dimensions is shown in figure 1*c*. Importantly, this pooling method has minimal pool overlap (pools share at most one common specimen). In the case of equal-sized pools for all dimensions, the number of specimens is $N_{s}=m^{d}$ and the number of tests is $N_{t}=d\cdot m^{d-1}$. Thus, pool size must be larger than the algorithm dimensionality to yield efficiency gains, as the number of tests required per specimen is χ=d/m.

We implemented this protocol with Monte Carlo simulations as a function of disease prevalence $\left(P_{D}=0.5-10\text{\%}\right)$, pool size (m=2−16) and dimensionality (d=3−4). For each set of simulation conditions, 10^3^–10^4^ hypercubes are generated and analysed. Each individual sample is randomly assigned a +/− state according to the average prevalence rate. To incorporate sensitivity loss due to pooling, a pool size-dependent fraction γ of the positive samples is designated as ‘undetectable’ and will not yield a true-positive test result upon pooling. Sensitivity loss is estimated using a large observational dataset of reverse transcription polymerase chain reaction (RT-PCR) cycle threshold numbers from positive clinical results (electronic supplementary material, figure S1). Pools that contain at least one detectable positive specimen yield a true-positive result with probability Se=95%; pools without a detectable positive specimen yield a false-positive result with probability 1−Sp=1%. For each hypercube, simulated test outcomes are used to compute the posterior probabilities for individual samples. The threshold for a positive screening outcome is set to a posterior probability of 40% or higher, selected on the basis of the ratio of known-positive and known-negative specimens at each posterior probability outcome in large-scale Monte Carlo simulations using the test characteristics detailed above (for details, see electronic supplementary material, figure S4 and accompanying text).

## 3. Results

### 3.1. Trade-off between efficiency, performance and complexity

**Figure 2.**
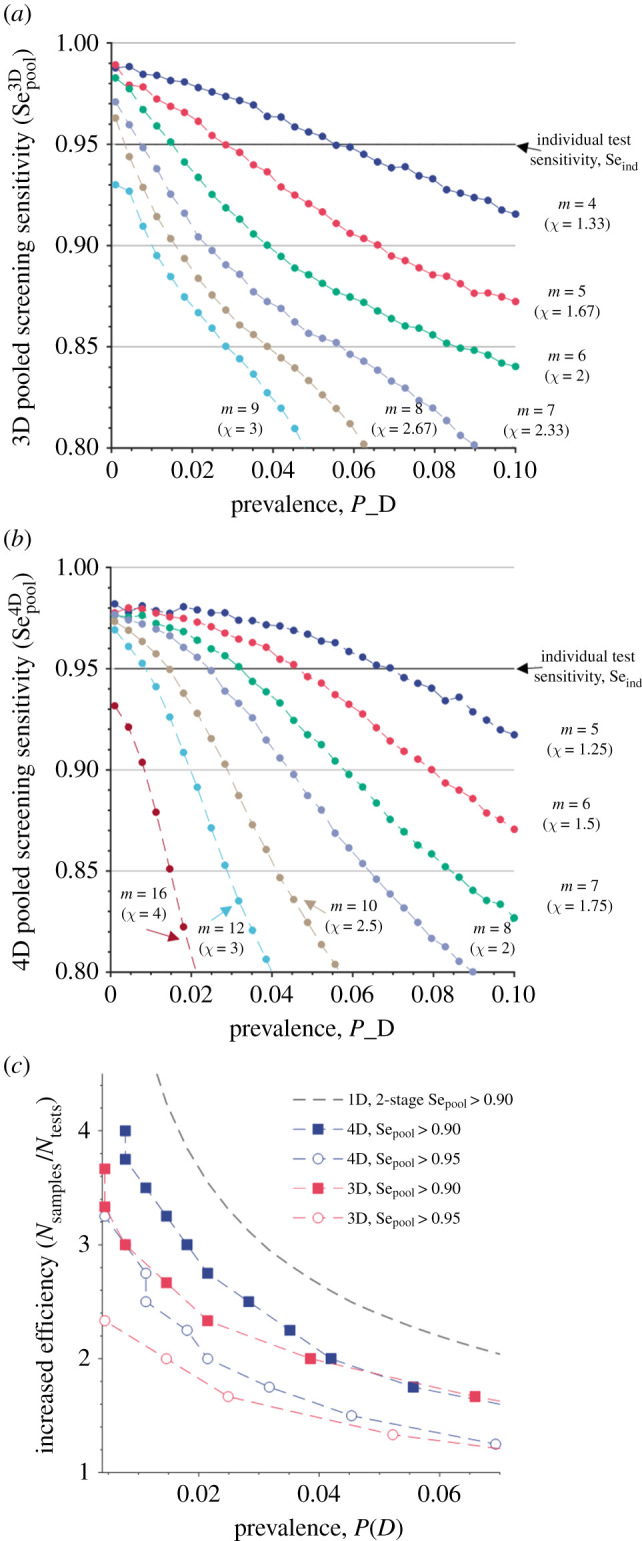
The detection sensitivity of a non-adaptive multi-dimensional pooled-sample screening as a function of disease prevalence in the screened population, for various pool sizes *m* and efficiency gains χ. (*a*) 3D pooling; (*b*) 4D pooling. The sensitivity of individual tests is Se=95%. (*c*) Screening efficiency (number of patients screened divided by the number of tests used) for 1D adaptive Dorfman pooling (grey dashed line) and non-adaptive multi-dimensional Bayesian screening in three and four dimensions is presented. For non-adaptive pooling, two sensitivity cut-off values were used: no sensitivity loss (circles) and 5% sensitivity loss (squares).

At low-to-moderate prevalence rates, it is possible to increase throughput without reducing the sensitivity or specificity of the pooled screening compared with the individual test (figure 2*a*,*b*). In fact, at low prevalence, this pooled testing protocol yields higher sensitivity than the individual test because the small number of positives leads to a low probability of overlap and each sample is included in *d* tests (d is the algorithm dimensionality). As long as the sample is not diluted below the detection limit, these multiple tests effectively reduce the chances of a false negative.

First, let us consider the prevalence range in which the pooled testing algorithm yields improved or equivalent test performance metrics. For a 3D implementation of the algorithm, a 33% increase in the sample throughput can be achieved for prevalence rates of up to 5% without any loss in sensitivity or specificity. For prevalence rates below 2%, the efficiency gains can be greater than a 2× factor without any reduction in performance. In a four-dimensional (4D) implementation, the higher algorithm dimensionality leads to an increase in the amount of information gathered via pooled testing at the cost of smaller efficiency gains: a 25% increase in efficiency, without performance losses, can be sustained up to an approximately 7% prevalence rate. An improvement in specificity is also observed, as expected for both adaptive and non-adaptive pooling (electronic supplementary material, figure S6).

As a comparison, we contrast the optimal screening efficiency achieved in our proposed method with that in a two-stage 1D adaptive pooling with overall sensitivity of 90% (figure 2*c*). This sensitivity reduction reflects the 95% agreement criteria between new and established protocols set forth by the US Food and Drug Administration for the Emergency Use Authorization of new molecular diagnostics [37], a stricter criterion than the 85% positive agreement for pooled testing [38]. For 3D and 4D pooling protocols that retain an overall sensitivity of ${\mathrm{Se}}_{\mathrm{pool}}\geq 90\text{\%}$, the throughput gains are smaller than those obtained with the adaptive 1D algorithm (e.g. at 2% prevalence the number of patients screened per test is 3.55, 2.33 and 2.75 for 1D, 3D and 4D, respectively). However, we note that it is possible for 3D and 4D pooled screening to retain the full sensitivity of an individual test and yield efficiency gains—at 2% prevalence, 1.67 and 2 patients are screened per test in 3D and 4D algorithms with ${\mathrm{Se}}_{\mathrm{pool}}\geq 95\text{\%}$, respectively. Supplementary Monte Carlo simulations focused on low prevalence and low viral load populations can be found in electronic supplementary material, figure S7.

### 3.2. Protocol validation for screening SARS-CoV-2 specimens

We implemented this protocol with 64 specimens initially screened as clinical samples at ARUP Laboratories. All samples were collected as nasopharyngeal swabs in phosphate-buffered saline (PBS) solution. Specimens were selected from the post-screening sample bank (frozen at −20°C) and were stripped of all protected health information. Three known positives were included in the pools, one of which (specimen 1) was flagged for serial dilutions (1×, 5× or 25× dilution in PBS) to explore the effects of dilution on test sensitivity. Besides specimen 1, all other specimens were randomly ordered (specimens 2–64). All specimens were individually retested in a control run, and 48 pools were prepared according to the previously described pooling arrangement with dimensionality d=3 and pool size m=4 (electronic supplementary material, tables S1–S2). Three repeat instances of the pooling arrangement were prepared, each one identical except for the serial dilution of specimen 1. Individual and pooled samples were tested using the Hologic Panther Fusion SARS-CoV-2 platform at ARUP Laboratories (outcomes in electronic supplementary material, tables S3–S6) [39]. Pooled test results were analysed with an automated Bayesian inference process whose output consists of the posterior probabilities for each individual specimen, and its only inputs are the estimated sensitivity loss due to pooling, the sensitivity and specificity of the individual test, and a list of pooled test results.

In the control run, two out of the three known-positive, undiluted specimens were correctly identified, while a known-positive sample yielded an invalid test result (test could not be repeated because of limited residual specimen volume). After a 5× dilution, specimen 1 was still detectable, and its cycle threshold number increased from $C_{t}=33.9$ to $C_{t}=36.5$ (for ideal-efficiency PCR, $\Delta C_{t}=\log_{2}(5)=2.3$). After 25× dilution, specimen 1 yielded a negative result in its control test. In addition, there were two samples whose initial clinical result was negative but yielded a positive result when tested as part of the control set (repeat tests were negative, although it is possible they are positives close to the limit of detection). We thus estimate the overall sensitivity in this control run as Se=3/5=60% (two undiluted + one diluted positives detected out of three undiluted + two diluted known positives), and the specificity as Sp=59/61=96.7% (two false positives out of 61 known negatives).

**Figure 3.**
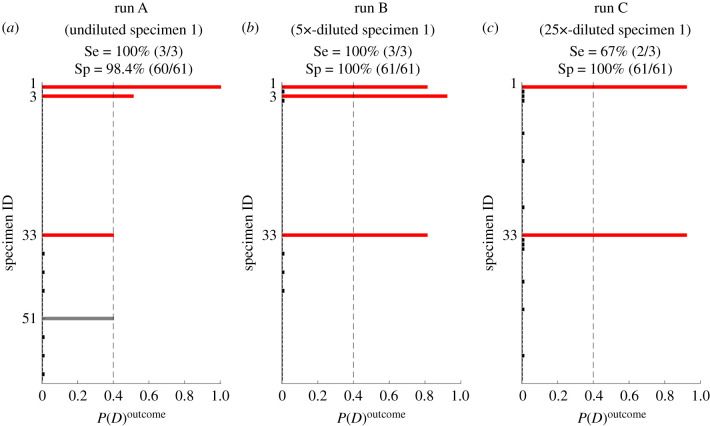
The posterior probabilities assigned to specimens in 3D pooling trials (run A, B and C in the corresponding figure panels). The vertical dashed line denotes the diagnosis threshold; specimens in red are known positives, grey are false positives. Observed sensitivity Se and specificity Sp in each screening run are noted in the figure.

Posterior probabilities for all samples in the pooled screening trials are shown in figure 3. In the pooled screening run whose inputs are undiluted specimens (figure 3*a*), all three known positives are correctly identified and one false positive is reported (100% sensitivity and 98.4% specificity). The pooled screening run which included a 5× dilution of specimen 1 yielded results with 100% sensitivity and specificity (figure 3*b*). Upon further dilution (25×) of specimen 1, the sensitivity of the pooled screening algorithm was reduced (Se=2/3=67%); no false positives were reported (figure 3*c*). These metrics are estimates based on a small number of tests and should be considered in the context of comparing the control run with the 3D pooling screening results.

## 4. Discussion

We present a pooled-sample testing methodology whose performance is explored with Monte Carlo simulations and validated experimentally, and show that it can retain high sensitivity even at moderate prevalence rates while also improving specificity and throughput. The increases in sensitivity and specificity gained by this multi-dimensional pooling strategy are the result of the inclusion of each sample in multiple tests and the minimal overlap between pooled samples. A Bayesian inference framework in which the assessed probability of positivity for each sample is updated several times allows the algorithm to effectively distinguish between positive and negative samples (electronic supplementary material, figures S2 and S3), which in turn enables a non-adaptive methodology—i.e. reporting of outcomes for both positive and negative specimens in one testing round without the need for retesting.

The experimental validation was performed manually, and thus the sample preparation burden was significant— setting up the individual tests was achieved in approximately 60 min versus approximately 210 min for their 48 pooled tests. While the actual sample preparation time is highly dependent on the user, it is clear that the additional effort precludes large-scale manual implementation. With regard to time to results, detailed accounting of the net efficiency gains will vary greatly not just because of sample preparation time, but will also depend on the test runtime and equipment modality (e.g. continuous sample loading versus batch processing). Importantly, because samples are pooled in predictable patterns and no post-screening sample retrieval is necessary, this protocol is compatible with automated sample processing (either bench- or laboratory-scale robotic liquid handlers). In fact, automation is critical for the long-term and high-volume implementation of this approach since it enables more complex versions (higher dimensionality and larger pool sizes) with larger efficiency gains and improved test performance, while eliminating human sources of error (e.g. cross-contamination) and reducing the additional burden of sample preparation.

Finally, it is important to consider the analysis of results and output generation. Upon implementation, the routine analysis of results requires no user input and can be automated; however, appropriate steps must be taken to interface its output into a format compatible with clinical laboratory information systems. Importantly, individual sample reports could be enhanced with a continuous variable—the posterior probability—and its interpretation.

To date, screening approaches based on pooling have found applications where efficiency in material resources is prioritized, but time is seldom a variable of interest, e.g. screening at blood banks. When applied at the population level (e.g. periodic prevalence monitoring), they are rarely expected to be deployed for an extended period of time or beyond a local scale. Thus, algorithms that are computationally complex or that require multiple rounds of testing have been favoured because they yield substantial gains in metrics such as samples screened per test used. However, as a result of the current SARS-CoV-2 pandemic, RT-PCR diagnostic testing has been carried out on large numbers of patients with sustained high demand—a situation that will likely intensify as communities worldwide move towards a reopening of economic activity and restrictions are lifted. Importantly, these testing efforts have a strong emphasis on quickly identifying positive cases in order to reduce community transmission—i.e. increasing the turnaround time for test results is not desirable. Additionally, the sensitivity and specificity characteristics of diagnostic tests are not perfect, and their availability may be limited. Under these novel constraints, pooled-sample screening protocols not only must increase sample throughput, but they should also minimize loss in sensitivity and maximize gains in specificity, while avoiding increases in the sample processing time resulting from repeated storage, retrieval and testing steps.

To address these limitations to scaling diagnostic testing for effective and sustained epidemiological surveillance at the population level in low-to-moderate prevalence groups, we propose a non-adaptive multi-dimensional pooling approach. Bayesian inference yields reportable outcomes at the individual specimen level in a single testing step with remarkable performance in a manner compatible with fully automated sample preparation and result interpretation. Considerable increases in sample throughput (greater than 2×) are achievable even at moderate prevalence rates (approx. 2%) without losses in test performance and without the need for multiple rounds of testing to reach individual-level outcomes for both positive and negative specimens. Accepting a moderate loss in screening sensitivity, in line with that of 1D pooling approaches, efficiency gains are larger and can be sustained at larger prevalence. With a combination of Monte Carlo simulations and a manual implementation with SARS-CoV-2 clinical samples, we demonstrate the viability of using this screening protocol in a real-world setting and contribute to solving the unprecedented challenges posed by the current SARS-CoV-2 pandemic.

## Data accessibility

All data are available in the main text or the supplementary materials. Code available via the online repository https://github.com/rnoriegam/bayesian-pooling. The data are provided in the electronic supplementary material.
